# Automated orofacial virtual patient creation using two cohorts of MSCT vs. CBCT scans

**DOI:** 10.1186/s13005-025-00500-1

**Published:** 2025-03-28

**Authors:** Thanatchaporn Jindanil, Oana-Elena Burlacu-Vatamanu, Benedetta Baldini, Joeri Meyns, Jeroen Meewis, Rocharles Cavalcante Fontenele, Maria Cadenas de Llano Perula, Reinhilde Jacobs

**Affiliations:** 1 OMFS-IMPATH Research Group, Department of Imaging and Pathology, Faculty of Medicine, KU Leuven, Leuven, Belgium; 2 Department of Oral and Maxillofacial Surgery, University Hospitals Leuven, Leuven, Belgium; 3 Department of Radiology, Faculty of Dentistry, Chulalongkorn University, Bangkok, Thailand; 4 Doctoral School, Carol Davila University of Medicine and Pharmacy, Bucharest, Romania; 5 Department of Electronics, Information and Bioengineering, Politecnico Di Milano, Milan, Italy; 6 UOC Maxillo-Facial Surgery and Dentistry Fondazione IRCCS Cà Granda, Ospedale Maggiore Policlinico, Milan, Italy; 7 Department of Oral and Maxillofacial Surgery, General Hospital St-Jan Genk, Genk, Belgium; 8 Department of Stomatology, Public Health and Forensic Dentistry, Division of Oral Radiology, School of Dentistry of Ribeirão Preto, University of São Paulo, Ribeirão Preto, Brazil; 9 Department of Oral Health Sciences - Orthodontics, KU Leuven and Dentistry, University Hospitals Leuven, Leuven, Belgium; 10 Department of Dental Medicine, Karolinska Institute, Stockholm, Sweden

**Keywords:** Virtual patient, Multimodal registration, Digital dentistry, Artificial intelligence, Convolutional neural network

## Abstract

**Background:**

Virtual simulation has advanced in dental healthcare, but the impact of different tomographic techniques on virtual patient (VP) creation remains unclear. This study primarily aimed to automatically create VP from facial scans (FS), intraoral scans (IOS), multislice (MSCT), and cone beam computed tomography (CBCT); Secondarily, to quantitatively compare artificial intelligence (AI)-driven, AI-refined and semi automatically registered (SAR) VP creation from MSCT and CBCT and to compare the effect of soft tissue on the registration with MSCT and CBCT.

**Methods:**

A dataset of 20 FS, IOS, and (MS/CB)CT scans was imported into the Virtual Patient Creator platform to generate automated VPs. The accuracy (percentage of corrections required), consistency, and time efficiency of the AI-driven VP registration were then compared to those of the AI-refined and SAR (clinical reference) using Mimics software. The surface distance between the registered FS and the (MS/CB)CT surface rendering using SAR and AI-driven methods was measured to assess the effect of soft tissue on registration.

**Results:**

All three registration methods achieved 100% accuracy for VP creation with both MSCT and CBCT (*p* > 0.999), with no significant differences between tomographic techniques either (*p* > 0.999). Perfect consistency (1.00) was obtained with AI-driven and AI-refined methods, and slightly lower for SAR (0.977 for MSCT and 0.895 for CBCT). Average registration times were 24.9 and 28.5 s for AI-driven and AI-refined, and 242.3 and 275.7 s for SAR with MSCT and CBCT respectively. The total time was significantly shorter for MSCT (313.7 s) compared to CBCT (850.3 s) (*p* < 0.001). While the average surface distance between MSCT- and CBCT-based VP showed no significant difference (*p* > 0.05), AI-driven resulted in a smaller surface distance than SAR (*p* < 0.05).

**Conclusions:**

AI enables fast, accurate, and consistent VP creation using FS, IOS, and (MS/CB)CT data. AI-driven, AI-refined, and semi-automated methods all achieve good accuracy. Additionally, soft tissue registration shows no significant difference between MSCT and CBCT.

**Supplementary Information:**

The online version contains supplementary material available at 10.1186/s13005-025-00500-1.

## Background

Virtual simulation has significantly advanced in dental healthcare. Over a decade has passed since Joda and Gallucci introduced the concept of the three-dimensional (3D) virtual patient (VP), the 3D model superimposed on the intraoral scan (IOS), extraoral scan or facial scan (FS), and cone beam computed tomography (CBCT) [1]. Each of these imaging devices is optimized to capture a different tissue type but currently no single imaging device can accurately capture them all, which is why the fusion of the imaging modalities is proposed as a tool for visualization and downstream function and analysis [2]. The model can be used to visualize and plan treatment strategies with remarkable precision and efficiency, for complex procedures such as dental restorations, orthodontic treatment, implant placement or maxillofacial surgery, while also improving communication with the patients and other healthcare professionals [3].

3D skeletal images can be obtained not only from CBCT but also from multislice computed tomography (MSCT) [4]. MSCT data acquisition uses a translate-and-rotate fan beam with a direct circular detector array to measure photon density with the patient in the supine position. The individual slices are then digitally post-processed and stacked together to create the 3D images [5, 6]. CBCT, which is more commonly used in dental and maxillofacial imaging, acquires volumetric data by simultaneously rotating a cone beam along with a two-dimensional (2D) detector array, typically with the patient in an upright position [6, 7]. MSCT is recognized for its superior image quality, particularly in terms of contrast resolution and noise level. However, CBCT can provide high-quality 3D images with lower radiation exposure and cost compared to MSCT [7–9]. The key distinction is that MSCT generates Hounsfield units, while CBCT produces quantitative gray values, which are not directly comparable and are primarily used in image processing for subsequent applications [10]. Artificial intelligence (AI) has demonstrated its potential to effectively overcome segmentation challenges for both MSCT and CBCT [11–16], being able to perform multimodal image registration with time efficiency, accuracy, and strong consistency [17, 18].

As all of the aforementioned processes are essential for VP, and despite the integration of dynamic occlusion and clinical space-time information to create four-dimensional models, the creation of VP still relies primarily on manual or semi-automated segmentation and registration techniques [19–21]. The integration of AI into this process could significantly enhance its development and provide an opportunity to finally incorporate the VP into daily practice, but there is a gap in understanding the possible impact of different tomographic techniques on VP creation. Thus, the primary objective of this study was to automatically create the VP from FS, IOS, and (MS/CB)CT. The secondary objectives were to quantitatively compare AI-driven, AI-refined, and semi-automatically registered VP from MSCT and CBCT and to compare the effect of soft tissue on MSCT and CBCT registration.

## Methods

### Aim

This study aimed to automate the creation of the VP using FS, IOS, and (MS/CB)CT data. Secondary objectives included comparing AI-driven, AI-refined, and semi-automatic VP registration methods for MSCT and CBCT, as well as assessing the impact of soft tissue on registration accuracy.

### Sample

The image dataset used in this study to create the VP belonged to 20 adolescents, 11 males (mean age 15.09 ± 2.07, range 12–20 years-old) and 9 females (mean age 14.78 ± 1.92, range 11–17 years-old), who were undergoing interceptive orthopedic orthodontic treatment with appliances for skeletal class II or III malocclusion in mixed dentition, both groups used a hybrid hyrax device in the upper jaw combined with either a removable facemask or a titanium mentoplate bone anchor in the mandible. The data was collected at the 5-year follow-up phase after the completion of their treatment.

Multimodal image data (MSCT, IOS, and FS) from 10 patients were used to create the VP model. These images were acquired during an ongoing randomized controlled trial taking place at Oost Limburg Hospital, Belgium (ethical approval numbers B3712201629565 and S67723) testing two types of interceptive orthopedic orthodontic appliances for skeletal class III malocclusion in mixed dentition. The sample included patients who returned for follow-up after interceptive treatment for correct cross-bite and Class III malocclusion. Ten additional multimodal image data (CBCT, IOS, and FS) of patients were acquired during an ongoing interceptive orthopedic orthodontic treatment for skeletal class II taking place at University Hospital Leuven, Belgium (ethical approval number S69363). These malocclusions were characterized by a class II molar relationship. Patients with a history of facial trauma, congenital anomalies, pathologies affecting facial contours, and poor-quality images such as blurring or excessive artifacts were excluded.

### Construction of the AI-driven, AI-refined and semiautomatic VP

3D FS images were acquired using the structured light-based scanner iReal 2E (Scantech, Hangzhou, China) according to the manufacturer’s guidelines. Post-processing of 3D images was performed using the scanner’s licensed software to capture the entire face and neck, including hair and ears. IOS images were obtained using the Emerald S scanner (Planmeca, Helsinki, Finland) and PANDA smart (Freqty Technology, Zhejiang, China). Low-dose MSCT scans were acquired using a SOMATOM Force scanner (SIEMENS, Munich, Germany) with an acquisition time of 5 s. The low-dose MSCT acquisition parameters were set at 150 kilovoltage-peak (kVp) and a tube current ranging from 49 to 199 milliamperes (mA). MSCT scan voxel sizes ranged from 0.39 × 0.39 × 0.60 mm^3^ to 0.49 × 0.49 × 0.60 mm^3^. CBCT scans were obtained with a NewTom VGi evo scanner (NewTom, Imola, Italy) with a scan time of 5 s. The CBCT acquisition parameters were set at 110 kVp with a tube current ranging from 4 to 8 mA and an acquisition time of 18 s with the voxel size of 0.30 × 0.30 × 0.30 mm^3^. Detailed (MS/CB)CT acquisition parameters are provided in Supplementary Table 1.

The files were saved in Object File Format (OBJ) for FS, Standard Tessellation Language (STL) for IOS, and Digital Imaging and Communication in Medicine (DICOM) for (MS/CB)CT scans. These files were uploaded to the Virtual Patient Creator (version 2.2.0, March 2022, Relu BV, Leuven, Belgium), an online cloud-based platform that enables automatic anatomical segmentation and registration (AI-driven) of the maxillofacial complex. The AI-driven segmentation of CBCT scans employs a validated 3D U-Net type convolutional neural network segmentation model, which incorporates convolutional layers with 4 encoding and 3 decoding blocks, each containing 2 convolutions, ReLU activation, group normalization, and 8 feature maps [14, 15, 22]. For IOS registration, the initial alignment of IOS and CBCT models is conducted using deep learning algorithms, followed by refinement with an Iterative Closest Point (ICP) approach. This process aligns segmented crowns from IOS with crowns from CBCT by optimizing transformations and iteratively minimizing alignment errors until convergence is achieved [23]. The registration of the FS is based on point prediction across imaging modalities, aligned with the segmented (MS/CB)CT. Deep learning algorithms are used to predict corresponding points on the IOS and FS, identifying these points based on shared anatomical or geometric features visible across all three modalities. This prediction process ensures spatial consistency of points across different imaging formats. Once the corresponding points are identified, the ICP algorithm is applied to minimize the distance between corresponding points across modalities, iteratively refining the alignment until an optimal match is achieved. The merged STL files of the three components or the VP were then exported into a single 3D STL model for subsequent evaluation.

Semi-automated registration (SAR) and refined automated registration (AI-refined) were performed using Mimics (version 24.0, Materialise N.V., Leuven, Belgium). For SAR, (MS/CB)CT scans were imported, and hard tissues, such as bones and teeth, were segmented. The IOS and FS were then registered using point-based registration to align the 3D images with the (MS/CB)CT segmented hard tissue or soft tissue contours. This process was followed by surface-based registration, with manual adjustments as needed. The process was conducted independently by two observers (T.J. and O.B.), who had comparable skills and experience in SAR, following prior calibration to ensure identical steps for both segmentation and registration.

### Quantitative comparison of VP registration methods: accuracy, consistency and time

The accuracy of each registration method was assessed by measuring the surface distance between the hard or soft tissue outlines on (MS/CB)CT scans and STL files in multiplanar reconstruction. Each registration was scored from 1 to 3 based on the maximum distance requiring correction (1: no correction (< 1 mm), 2: minor correction (1–3 mm), 3: major correction needed (> 3 mm)). Separate scores were given for the upper, middle, and lower thirds of the face. Additionally, discrepancies among FS, IOS, and (MS/CB)CT in these three areas were noted.

To evaluate the consistency of the AI-driven VP, FS, IOS, and (MS/CB)CT scans were imported and exported twice from the online platform. For the AI-refined and SAR methods, two observers (T.J. and O.B.) performed a second registration session two weeks after the initial registration to assess both inter- and intra-observer reliability. The same methodology used to assess the accuracy was used to assess consistency.

The time required for each registration method was recorded in seconds. For the AI-driven VP creation, the timing started when the DICOM file was opened in the Virtual Patient Creator platform and ended when the complete VP model was generated. For the AI-refined method, the time was recorded in addition to the AI-driven method, starting from the import of the integrated STL files until VP model refinement was completed. For the SAR method, both observers (T.J. and O.B.) recorded the time from hard tissue segmentation to VP creation. The mean registration and total time from both observers were used to represent SAR method. Additionally, segmentation time was recorded to calculate the total time involved.

### Comparison of MSCT and CBCT soft tissue impact on the registration

The impact of soft tissue on patient positioning in MSCT and CBCT was evaluated by comparing the soft tissue surface rendering model from the tomographic scans with the FS. Soft tissue rendering was processed in the Mimics software (version 24.0, Materialise, Leuven, Belgium) using predefined soft tissue threshold settings (Hounsfield unit: -700 to 225) for MSCT, while an adjusted grayscale setting was used for CBCT. For both CBCT and MSCT groups, the point-to-point surface distance in 3D space was measured between the registered FS, aligned using either SAR or AI-driven registration methods, and the corresponding surface rendering for each case. In addition, the surface rendering and FS from both registration methods were superimposed and cropped into three different facial regions: upper (from the trichion to nasion landmarks), middle (from the nasion to subnasale landmarks), and lower (from the subnasal to menton landmarks). The surface distance between the surface rendering and the FS was also calculated for each facial region.

**Fig. 1:**
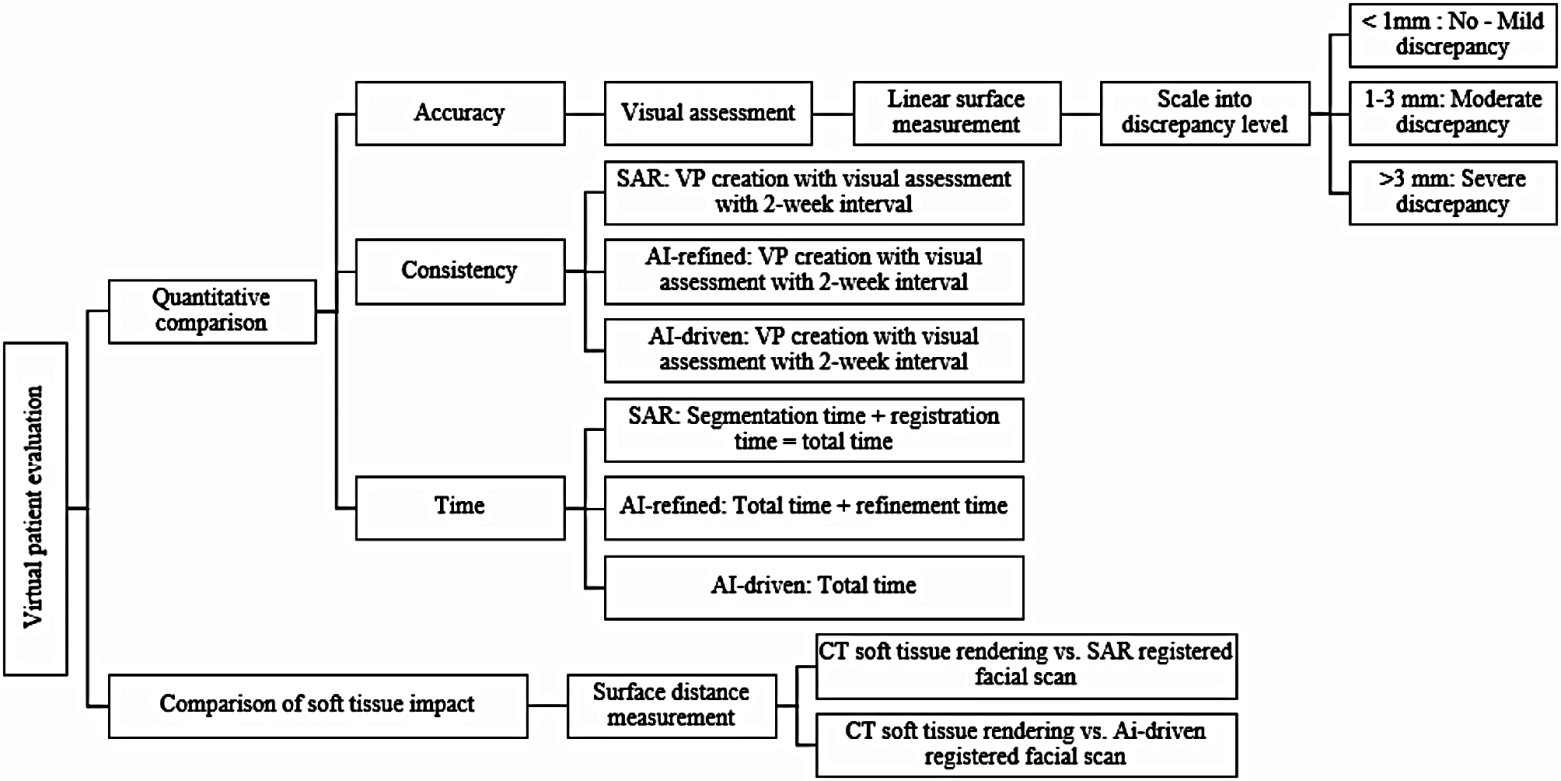
Flowchart of the virtual patient evaluation procedures

Figure 1 presents a flowchart outlining the virtual patient evaluation procedures.

### Statistical analysis

To analyze accuracy, the normality of the data distribution was first tested using Shapiro-wilk test. Since the data were not normally distributed, the accuracy results of the AI-driven, AI-refined, and SAR methods were statistically compared using the Kruskal-Wallis test, followed by post-hoc analysis with Bonferroni correction. Time differences between the three registration methods were analyzed using one-way analysis of variance (ANOVA), while the differences between the two observers performing SAR were assessed by independent samples t-test. Additionally, a paired sample t-test was used to evaluate the consistency of time recordings for each method by comparing the AI-driven, AI-refined, and SAR methods in pairs.

To evaluate registration consistency, intra- and inter-observer reliability were calculated for each registration method. Test-retest reliability was assessed using the Spearman correlation coefficient (SCC). Reliability results were interpreted according to the criteria of Schober et al.: 0-0.1 indicates negligible correlation; 0.1–0.39 indicates weak correlation; 0.4–0.69 indicates moderate correlation; 0.7–0.89 indicates strong correlation; and 0.9-1.00 indicates very strong correlation [24].

For the surface distance analysis, the Shapiro-Wilk test was also used to assess data distribution normality. Since non-normal distribution was confirmed, Wilcoxon matched-pair signed-rank test was used to evaluate the distance differences between SAR and AI-driven registration methods. Friedman’s test was applied to assess differences within the same tomographic technique for each facial region. The Wilcoxon signed-rank test was used to compare different tomographic techniques.

## Results

### Quantitative comparison of VP registration methods: accuracy, consistency and time

As shown in Table 1, no corrections were needed for either VP with MSCT or CBCT in the upper and middle thirds of the face, indicating 100% accurate registration for all three registration methods (*p* > 0.999). However, in the lower facial third, 30% of the VP created with MSCT required no corrections, 60% needed minor corrections, and 10% needed major corrections (*p* > 0.999). In contrast, VP with CBCT had more inaccuracies in the lower facial third, with 20% requiring no corrections, and 40% needing minor and major corrections for all registration methods (*p* > 0.999). There was no statistically significant difference in registration accuracy among AI-driven, AI-refined, and SAR VP methods for both MSCT and CBCT (*p* > 0.999), nor between VP with MSCT or VP with CBCT (*p* > 0.999). In addition, registration between the IOS and (MS/CB)CT was 100% correct for all three registration methods, with no statistically significant differences observed (*p* > 0.999).

The registration consistency is also shown in Table 1. The consistency for all registration methods was relatively high, with AI-driven and AI-refined registrations being the highest for both VP with MSCT and CBCT (SCC = 1, *p* < 0.01). The consistency of SAR was higher for VP with MSCT (intra-observer SCC = 0.997-1, *p* < 0.01; inter-observer SCC = 0.997, *p* < 0.01) compared to CBCT (intra-observer SCC = 0.757-1, *p* < 0.01; inter-observer SCC = 0.895, *p* < 0.01).

For cases with MSCT, the average registration time and total time for AI-driven and AI-refined methods were both 24.9 s (*p* > 0.999), while SAR required 242.3 s for registration (*p* < 0.001), with a total time of 313.7 s. For cases with CBCT, the average registration time and total time for the AI-driven and AI-refined methods were 28.5 s, showing no statistically significant difference from the MSCT cases (*p* > 0.5). However, the SAR method for CBCT cases had a registration time of 275.7 s and a total time of 850.3 s, which is statistically significantly longer compared to those with MSCT (*p* < 0.001). When comparing the time between two observers, a statistically significant difference was found only in the total time for CBCT (*p* < 0.05) (Table 2).

### Comparison of MSCT and CBCT soft tissue impact on the registration

**Fig. 2:**
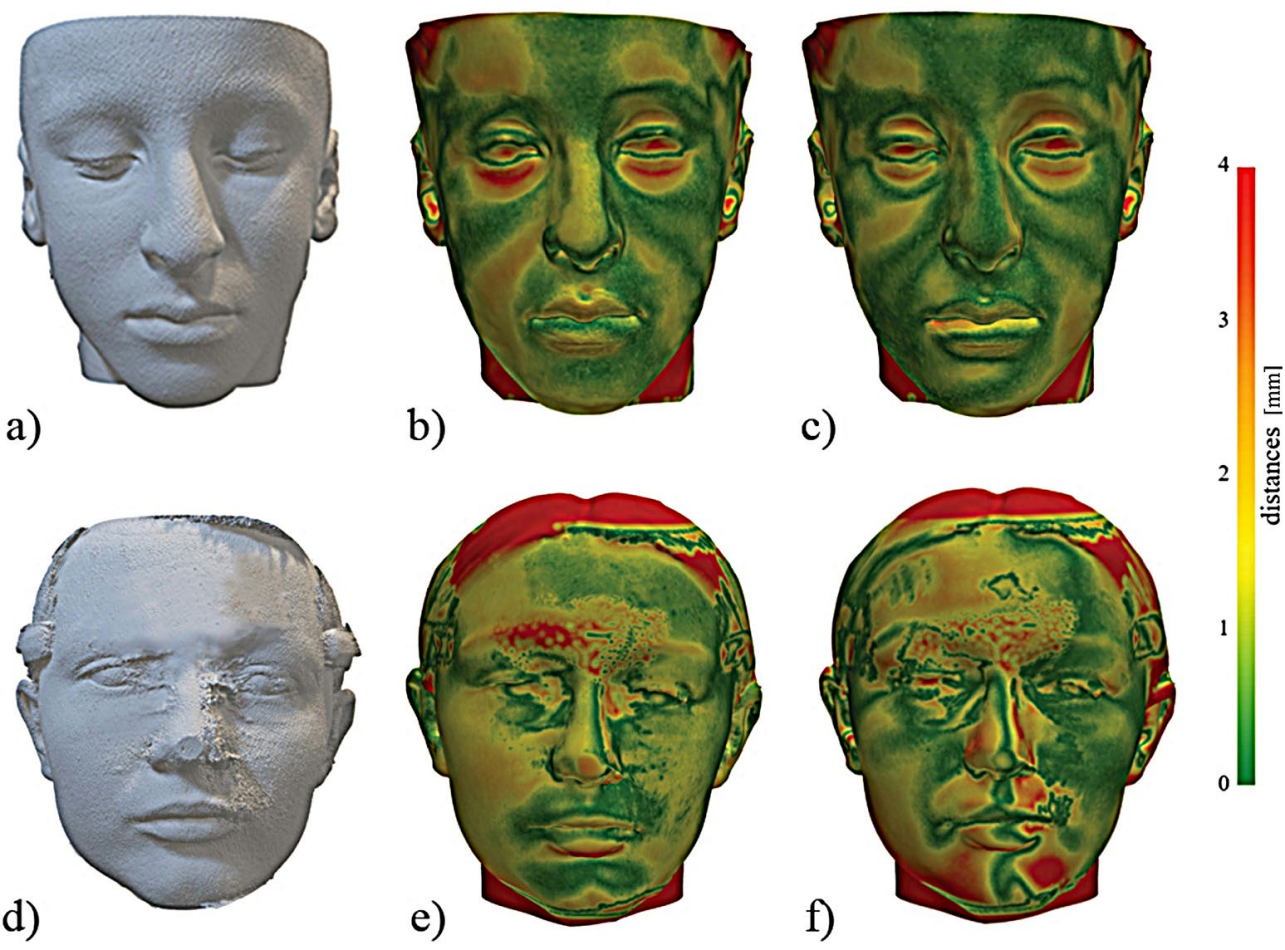
Distance map between surface rendering and registered facial scan **a**) Multislice computed tomography (MSCT) surface rendering **b**) Distance map between automatically registered facial scan and MSCT surface rendering **c**) Distance map between semi-automatically registered facial scan and MSCT surface rendering **d**) Cone beam computed tomography (CBCT) surface rendering **e**) Distance map between automatically registered facial scan and CBCT surface rendering **f**) Distance map between semi-automatically registered facial scan and CBCT surface rendering, A-C is a 14-year old male patient, D-F is a 15-year old female patient

There was no statistically significant difference in the average surface distance between the AI-driven and SAR registered FS, and the surface rendering obtained from both MSCT and CBCT (*p* > 0.05). When comparing the surface distance across the three facial regions, no statistically significant differences were found for MSCT for both AI-driven and SAR registration methods, and for CBCT with the AI-driven registration method. However, significant differences were observed in the lower third of the face compared to other facial regions for the VP with CBCT using the SAR method (*p* < 0.05) (Fig. 2, Supplementary Table 2).

**Fig. 3:**
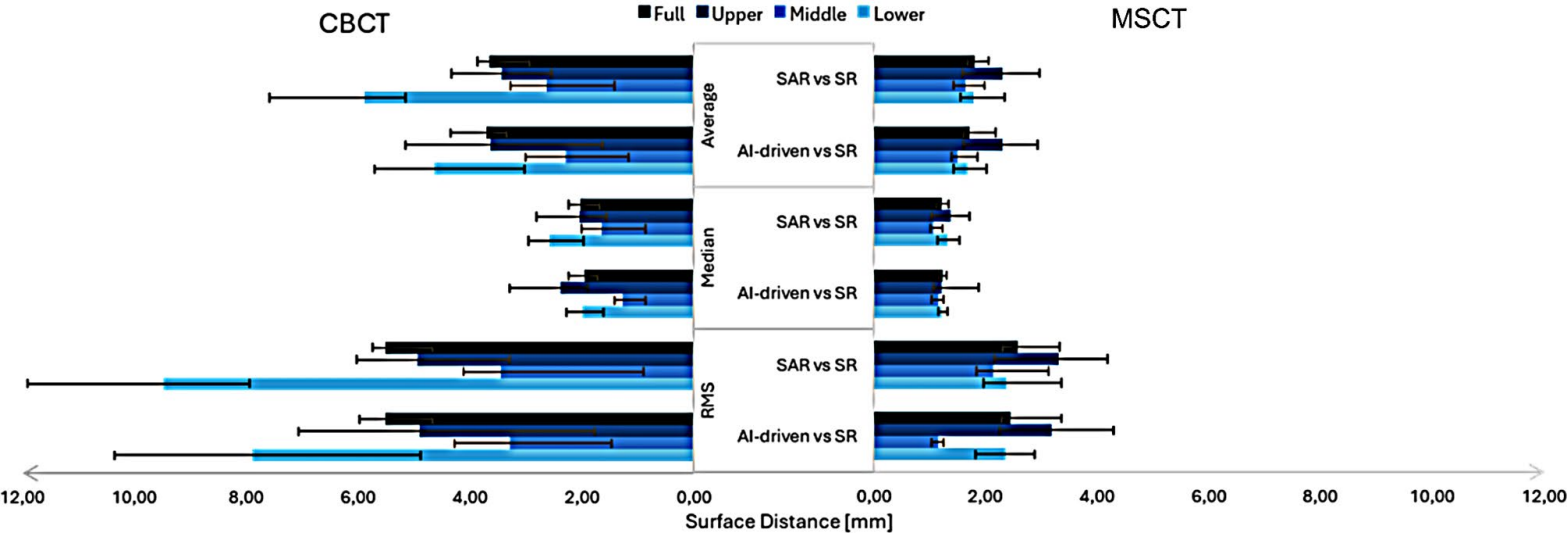
Surface distance between (cone beam) computed tomography surface rendering and registered facial scan MSCT: Multislice computed tomography, CBCT: Cone beam computed tomography, AI: Artificial intelligence, SAR: Semi-automated registration, SR: Surface rendering, RMS: Root mean square error

Considering the distances between the two tomographic scans, MSCT showed a smaller average distance to surface rendering compared to CBCT. For the entire face, AI-driven registered FS provided a smaller distance to surface rendering than SAR (*p* < 0.001). In the upper facial region, there was no statistically significant difference between MSCT and CBCT surface rendering with the registered FS from both registration methods (*p* = 0.218). However, a difference was found in the middle third of the face for the SAR methods (*p* = 0.07), indicating a greater distance with SAR-registered FS and CBCT surface rendering compared to MSCT. Significant differences were also observed in the lower facial regions for both registration methods, following the same trend as the whole full face, with MSCT showing a smaller average distance compared to CBCT and AI-driven registered FS providing a smaller distance to surface rendering than SAR (*p* < 0.001) (Fig. 3, Supplementary Table 2).

**Table 1:** Accuracy and consistency of AI-driven, AI-refined and semi-automatically registered orofacial virtual patient per facial region

Registration mode	% Correction needed																					Consistency		*P* value CT vs. CBCT
	Upper facial third							Middle facial third							Lower facial third									
	CT			CBCT				CT			CBCT				CT			CBCT				CT	CBCT	
	No	Minor	Major	No		Minor	Major	No	Minor	Major	No		Minor	Major	No	Minor	Major	No		Minor	Major			
AI-driven	100	0	0	100		0	0	100	0	0	100		0	0	30	60	10	20		40	40	1	1	> 0.999
AI-refined	100	0	0	100		0	0	100	0	0	100		0	0	30	60	10	20		40	40	1	1	> 0.999
SAR	100	0	0	100		0	0	100	0	0	100		0	0	30	60	10	20		40	40	0.997	0.895	> 0.999
	P value							P value							P value									
	CT				CBCT			CT				CBCT			CT				CBCT					
AI-driven vs. SAR	> 0.999				> 0.999			> 0.999				> 0.999			> 0.999				> 0.999					
AI-refined vs. SAR	> 0.999				> 0.999			> 0.999				> 0.999			> 0.999				> 0.999					
AI-driven vs. AI refined	> 0.999				> 0.999			> 0.999				> 0.999			> 0.999				> 0.999					

AI: Artificial intelligence, SAR: Semi-automated registration, CT: Computed tomography, CBCT: Cone beam computed tomography, vs.: versus

Note that no correction needed = < 1 mm, Minor correction needed = 1–3 mm, Major correction needed = > 3 mm

**Table 2:** Comparison of the time required per registration method

Registration methods	MSCT		CBCT		*P* value MSCT vs. CBCT	
	Registration time (s)	Total time (s)	Registration time (s)	Total time (s)	Registration time (s)	Total time (s)
AI-driven	24.9 *±* 6.3	24.9 *±* 6.3	28.5 *±* 4.6	28.5 *±* 4.6	0.513	0.513
AI-refined	24.9 *±* 6.3	24.9 *±* 6.3	28.5 *±* 4.6	28.5 *±* 4.6	0.513	0.513
Average SAR	242.3 *±* 33.5	313.7 *±* 32.3	275.7 *±* 60.3	850.3 *±* 174.0	< 0.001	< 0.001
SAR Observer 2	232.7 *±* 30.1	292.2 *±* 36.3	242.8 *±* 101.8	635.3 *±* 134.7		
SAR Observer 1	251.9 *±* 55.8	335.2 *±* 53.1	308.5 *±* 53.9	1065.3 *±* 407.6		
	P value	P value	P value	P value		
AI-driven vs. SAR	< 0.001	< 0.001	< 0.001	< 0.001		
AI-refined vs. SAR	< 0.001	< 0.001	< 0.001	< 0.001		
AI-driven vs. AI-refined	> 0.999	> 0.999	> 0.999	> 0.999		
Observer 1 vs. Observer 2	0.334	0.063	0.090	0.023		

AI: Artificial intelligence, SAR: Semi-automated registration, MSCT: Multislice computed tomography, CBCT: Cone beam computed tomography, vs.: versus, s: Seconds

## Discussion

This study is the first to compare the accuracy and efficiency of different tomographic techniques, specifically MSCT and CBCT, in the context of automated VP creation, both for quantitative comparison and for the effect of soft tissue on the registration of different imaging modalities. AI showed good performance in terms of accuracy, consistency, and time-efficiency in both cases, regardless of the tomographic imaging modality, proving to be a possible alternative method for creating VP.

Quantitative comparison showed that all three registration methods could provide clinically acceptable registration accuracy in the upper and middle thirds of the face for both tomographic imaging modalities. However, more inaccuracies were found in the lower third, particularly in the VP with CBCT, which required more correction than those with MSCT. This discrepancy may be due to differences in patient positioning. Patients undergoing MSCT scans are positioned supine with a wax bite to obtain optimal occlusion. Therefore, not only gravity but also muscle tension could affect the lower facial third. In contrast, both CBCT and FS were acquired in an upright position. The fixation component of CBCT, such as the chin rest used for the patients in this study, can influence the soft tissue profile in the lower third of the face, as seen in Supplementary Fig. 1. However, the difference in accuracy between the VP created with the two tomographic imaging modalities was not statistically significant, indicating that the effect of gravity may not significantly alter the soft tissue position.

While this study is novel in investigating the accuracy of the virtual patient, previous studies have validated various steps in the creation of the virtual patient, such as the segmentation of anatomical structures and the registration process, showing high levels of accuracy. However, the integration with the FS has not been investigated until now. Considering the registration methods, AI can create VP as accurately as SAR, but with perfect consistency regardless of different tomographic scans, while the consistency of VP created with SAR was better with MSCT. These findings highlight the advantages of AI-driven methods in reducing human-induced variability and providing more reliable, repeatable results, consistent with the previous AI studies [14–18, 22].

Another important clinical finding from this study was the time efficiency of the AI-driven method compared to the current clinical standard SAR approach, despite requiring some corrections. While the average registration time using MSCT and CBCT was similar, there was a notable difference in the total time required, likely due to variations in segmentation times. This may be due to a clear segmentation threshold of a standardized Hounsfield unit for MSCT, which allows for precise and consistent segmentation. In contrast, CBCT relies on grey values, which are relative and cannot be standardized, leading to grey values variability and increased noise due to factors like scatter and beam hardening, which can result in lower tissue contrast [10, 24]. The conversion of these greyscales into pseudo-units can complicate the segmentation process, making it more challenging to use VP in daily practice due to the process complexity. In the current study, the semi-automated segmentation based on the designated Hounsfield threshold resulted in more artifacts in the hard tissue model segmented from the CBCT compared to the MSCT model. While the voxel size of CBCT in this study is smaller than that of MSCT, which theoretically should lead to a more precise segmentation model, CBCT is more susceptible to inherent noise. When combined with the quantitative gray values of the CBCT which are not calibrated according to Hounsfield unit, unlike MSCT, the semi-automated segmentation is better to MSCT than CBCT. The semi-automated segmentation approach on CBCT introduces artifacts that create additional challenges during the registration process by obscuring portions of the teeth. Nonetheless, it is worth noting that these artifacts had no impact on the registration accuracy nor on the registration time of both AI-driven and SAR approached. This aligns with the findings of previous study by Buin et al. [25], which confirm the feasibility of CBCT and IOS registration, even in the presence of artifacts, with discrepancies of less than 0.5 mm. The current results support the findings of several previous AI-based studies, showing that AI can overcome these complexities and provide fast, accurate, and consistent segmentation, regardless of the tomographic imaging modalities [22, 26–29]. When combined with registration, AI could offer a feasible solution for integrating VP into routine clinical workflows. This will not only support personalized diagnosis and treatment planning with greater precision, but it also has the potential to enhance applications in dental education and to improve patient communication and satisfaction.

When considering the effect of soft tissue on registration, it was expected that gravity would affect the soft tissue [30, 31]. However, the difference in surface distance between the AI-driven and SAR methods was not significant when comparing VP with MSCT and CBCT, suggesting that gravity did not have a clear effect on soft tissue registration. Similarly, as described by Yokoyama et al., gravity does not appear to significantly impact brain structure [32]. The SAR method exhibited greater variability in surface distance, especially in the middle and lower thirds of the face, particularly with CBCT. This finding highlights the importance of the reduction in registration variability that AI could provide.

The main limitation of this study is the limited size and non-homogeneity of the sample, as the MSCT and CBCT samples are not identical. However, achieving a fully homogeneous patient group is challenging due to radiation protection concerns. The selected patients were of relative same age and had comparable occlusal conditions. A larger sample size including patients with varying skeletal classifications in future studies would provide more insightful results on this topic. Despite these limitations, this study presents an innovative solution for clinicians to seamlessly integrate VP into daily practice. By comparing various methods for generating VP, this study ensures that the most accurate and reproducible approach is identified, which is essential for widespread adoption in clinical practice. Moreover, the AI-driven methods for VP construction not only ensure accuracy and consistency, but also reduce human-induced variability, making them a practical choice for routine clinical use.

## Conclusions

AI enables fast, accurate, and consistent VP creation through multimodal registration of hard and intra-oral as well as extra-oral soft tissues. AI-driven, AI-refined, and SAR methods can all achieve high accuracy in creating VP using either MSCT or CBCT, though CBCT tends to show more discrepancies. Additionally, soft tissue registration showed no significant differences between MSCT and CBCT, suggesting both are equally reliable for VP.

## Electronic supplementary material

Below is the link to the electronic supplementary material.

Supplementary Material 1

Supplementary Material 2

## Data Availability

No datasets were generated or analysed during the current study.

## Abbreviations

2D: Two-dimensional
3D: Three-dimensional
AI: Artificial intelligence
CBCT: Cone beam computed tomography
DICOM: Digital Imaging and Communication in Medicine
FS: Facial scan
ICP: Iterative Closest Point
IOS: Intraoral scan
kVp: Kilovoltage-peak
mA: Milliampere
MSCT: Multislice computed tomography
OBJ: Object File Format
SAR: Semi automatically registered
STL: Standard Tessellation Language
VP: Virtual patient
