# Supplementary material for: Automated orofacial virtual patient creation using two cohorts of MSCT vs. CBCT scans
Source: Head Face Med. 2025 Mar 28;21:21. doi: 10.1186/s13005-025-00500-1 (PMC11951535; doi:10.1186/s13005-025-00500-1)
Supplement: Supplementary file 1 — Supplementary Material 1 [file 13005_2025_500_MOESM1_ESM.docx]

**Supplementary Table 1.** (Multislice/Cone beam) computed tomography acquisition parameters

| **Acquisitionmode** | **Patient** | **Sex** | **Age** | **kVp** | **Tube current**  **(mA)** | **Acquisition time**  **(s)** | **Voxel size (mm^3^)** |
| --- | --- | --- | --- | --- | --- | --- | --- |
| MSCT | 1 | Male | 15 | 150 | 53 | 5 | 0.45 x 0.45 x 0.60 |
| MSCT | 2 | Male | 14 | 150 | 116 | 5 | 0.46 x 0.46 x 0.60 |
| MSCT | 3 | Female | 17 | 150 | 44 | 5 | 0.45 x 0.45 x 0.60 |
| MSCT | 4 | Male | 15 | 150 | 49 | 5 | 0.49 x 0.49 x 0.60 |
| MSCT | 5 | Female | 17 | 150 | 93 | 5 | 0.49 x 0.49 x 0.60 |
| MSCT | 6 | Female | 14 | 150 | 199 | 5 | 0.47 x 0.47 x 0.60 |
| MSCT | 7 | Male | 15 | 150 | 115 | 5 | 0.43 x 0.43 x 0.60 |
| MSCT | 8 | Female | 15 | 150 | 48 | 5 | 0.39 x 0.39 x 0.60 |
| MSCT | 9 | Male | 12 | 150 | 186 | 5 | 0.43 x 0.43 x 0.60 |
| MSCT | 10 | Male | 13 | 150 | 175 | 5 | 0.47 x 0.47 x 0.60 |
| CBCT | 11 | Female | 15 | 110 | 7 | 18 | 0.30 x 0.30 x 0.30 |
| CBCT | 12 | Female | 11 | 110 | 5 | 18 | 0.30 x 0.30 x 0.30 |
| CBCT | 13 | Male | 16 | 110 | 5 | 18 | 0.30 x 0.30 x 0.30 |
| CBCT | 14 | Male | 14 | 110 | 6 | 18 | 0.30 x 0.30 x 0.30 |
| CBCT | 15 | Male | 20 | 110 | 6 | 18 | 0.30 x 0.30 x 0.30 |
| CBCT | 16 | Female | 13 | 110 | 4 | 18 | 0.30 x 0.30 x 0.30 |
| CBCT | 17 | Male | 16 | 110 | 6 | 18 | 0.30 x 0.30 x 0.30 |
| CBCT | 18 | Female | 15 | 110 | 4 | 18 | 0.30 x 0.30 x 0.30 |
| CBCT | 19 | Female | 16 | 110 | 4 | 18 | 0.30 x 0.30 x 0.30 |
| CBCT | 20 | Male | 16 | 110 | 8 | 18 | 0.30 x 0.30 x 0.30 |

MSCT: Multislice computed tomography, a wax bit was in position during image acquisition of MSCT, CBCT: Cone beam computed tomography

**Supplementary table 1.** Surface distance between (multislice/cone beam) computed tomography surface rendering and registered facial scan

| Regions | MSCT | | | | | |  | CBCT | | | | | | | P value MSCT vs.CBCT | |
| --- | --- | --- | --- | --- | --- | --- | --- | --- | --- | --- | --- | --- | --- | --- | --- | --- |
|  | AI-driven FS vs. SR | | | SAR FS vs. SR | | | P value AI-driven vs. SAR | AI-driven FS vs. SR | | | SAR FS vs. SR | | | P value AI-driven vs. SAR | AI-driven | SAR |
|  | Avg | Med | RMS | Avg | Med | RMS |  | Avg | Med | RMS | Avg | Med | RMS |  |  |  |
| Full |  |  |  |  |  |  | 0.646 |  |  |  |  |  |  | 0.285 | <0.001 | <0.001 |
| Q1 | 1.61 | 1.16 | 2.29 | 1.67 | 1.12 | 2.31 |  | 3.08 | 1.66 | 5.06 | 3.43 | 1.82 | 5.28 |  |  |  |
| Median | 1.71 | 1.24 | 2.44 | 1.79 | 1.21 | 2.57 |  | 3.71 | 1.95 | 5.52 | 3.65 | 2.03 | 5.51 |  |  |  |
| Q3 | 2.18 | 1.30 | 3.35 | 2.06 | 1.34 | 3.33 |  | 4.07 | 2.18 | 6.35 | 4.36 | 2.38 | 6.35 |  |  |  |
| Min | 1.44 | 1.01 | 1.86 | 1.45 | 1.06 | 1.94 |  | 2.4 | 1.41 | 3.87 | 3.28 | 1.54 | 5.06 |  |  |  |
| Max | 3.15 | 1.91 | 4.68 | 3.07 | 1.7 | 4.61 |  | 5.76 | 3.63 | 8.43 | 6.53 | 3.58 | 10.15 |  |  |  |
| Upper |  |  |  |  |  |  | 0.285 |  |  |  |  |  |  | 0.386 | 0.218 | 0.218 |
| Q1 | 1.61 | 1.06 | 2.24 | 1.58 | 1.03 | 2.17 |  | 2.12 | 1.48 | 2.76 | 2.57 | 1.27 | 3.84 |  |  |  |
| Median | 2.31 | 1.21 | 3.18 | 2.30 | 1.37 | 3.32 |  | 3.64 | 2.39 | 4.91 | 3.45 | 2.04 | 4.93 |  |  |  |
| Q3 | 2.94 | 1.88 | 4.30 | 2.97 | 1.71 | 4.19 |  | 5.64 | 2.86 | 8.04 | 4.35 | 2.52 | 6.56 |  |  |  |
| Min | 1.31 | 0.83 | 1.79 | 1.18 | 0.76 | 1.63 |  | 1.45 | 1.14 | 1.89 | 1.16 | 0.90 | 1.47 |  |  |  |
| Max | 6.76 | 6.53 | 8.26 | 6.415 | 5.69 | 8.14 |  | 9.14 | 7.41 | 11.98 | 5.34 | 4.05 | 9.16 |  |  |  |
| Middle |  |  |  |  |  |  | 0.271 |  |  |  |  |  |  | 0.093 | 0.089 | 0.007 |
| Q1 | 1.39 | 1.03 | 1.03 | 1.43 | 1.02 | 1.85 |  | 1.56 | 1.15 | 2.31 | 2.00 | 1.30 | 2.78 |  |  |  |
| Median | 1.50 | 1.16 | 1.16 | 1.65 | 1.06 | 2.14 |  | 2.29 | 1.28 | 3.29 | 2.63 | 1.66 | 3.45 |  |  |  |
| Q3 | 1.86 | 1.25 | 1.25 | 1.99 | 1.23 | 3.13 |  | 3.40 | 1.71 | 5.11 | 3.84 | 2.44 | 5.99 |  |  |  |
| Min | 1.32 | 0.92 | 1.69 | 1.15 | 0.79 | 1.54 |  | 1.47 | 0.97 | 2.02 | 1.71 | 1.18 | 2.44 |  |  |  |
| Max | 3.16 | 1.57 | 5.2 | 2.89 | 1.36 | 5.04 |  | 3.63 | 1.99 | 5.82 | 7.27 | 3.48 | 11.45 |  |  |  |
| Lower |  |  |  |  |  |  | 0.445 |  |  |  |  |  |  | 0.241 | <0.001 | <0.001 |
| Q1 | 1.43 | 1.16 | 1.81 | 1.56 | 1.14 | 1.97 |  | 3.59 | 1.72 | 5.42 | 4.20 | 2.19 | 7.03 |  |  |  |
| Median | 1.68 | 1.21 | 2.37 | 1.78 | 1.31 | 2.37 |  | 4.64 | 2.00 | 7.89 | 5.89 | 2.58 | 9.47 |  |  |  |
| Q3 | 2.01 | 1.32 | 2.88 | 2.34 | 1.54 | 3.36 |  | 6.27 | 2.38 | 10.88 | 6.62 | 3.18 | 11.01 |  |  |  |
| Min | 1.37 | 1.06 | 1.69 | 1.28 | 1.04 | 1.56 |  | 2.09 | 1.33 | 3.14 | 2.90 | 1.39 | 3.86 |  |  |  |
| Max | 3.01 | 2.63 | 3.98 | 2.81 | 2.20 | 3.75 |  | 7.55 | 4.68 | 11.93 | 7.40 | 4.47 | 11.52 |  |  |  |
|  | P value | | | P value | | |  | P value | | | P value | | |  |  |  |
| U vs. M | 0.076 | | | 0.791 | | |  | >0.999 | | | 0.799 | | |  |  |  |
| M vs. L | 0.791 | | | >0.999 | | |  | 0.076 | | | 0.028 | | |  |  |  |
| U vs. L | 0.791 | | | >0.999 | | |  | 0.438 | | | 0.007 | | |  |  |  |

AI: Artificial intelligence, SAR: Semi-automated registration, MSCT: Multislice computed tomography, CBCT: Cone beam computed tomography, vs.: versus, Avg: Average, Q: Quartile, Min: Minimum, Max: Maximum, U: Upper, M: Middle, L: Lower


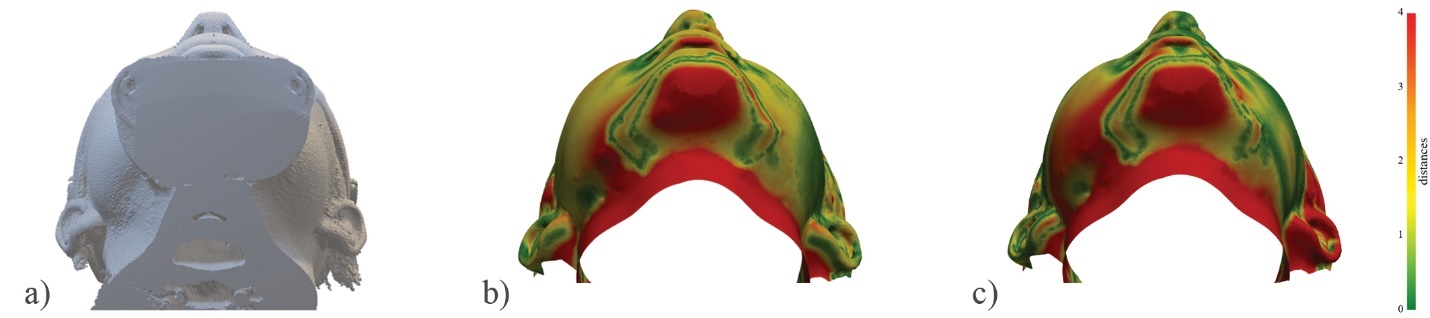


[mm]

**Supplementary figure 1.** Distance map at area under the chin of example Cone beam computed (CBCT) case a) CBCT surface rendering b) Distance map between automatically registered facial scan and CBCT surface rendering c) Distance map between semi-automatically registered facial scan and CBCT surface rendering
